# Supplementary material for: Resolving the full spectrum of human genome variation using Linked-Reads
Source: Genome Res. 2019 Apr;29(4):635–45. doi: 10.1101/gr.234443.118 (PMC6442396; doi:10.1101/gr.234443.118)
Supplement: Supplemental Material [file supp_gr.234443.118_Supplemental_longranger-2.2.zip › longranger-2.2.tar.bz2!/lr2.2-public/lib/stan/cmdstan/stan/doc/api/html/classstan_1_1gm_1_1singleton__argument.html]

Stan: stan::gm::singleton\_argument< T > Class Template Reference


|  |  |
| --- | --- |
|  | Stan  2.5.0  probability, sampling & optimization |


- Main Page
- Related Pages
- Namespaces
- Classes
- Files

- Class List
- Class Index
- Class Hierarchy
- Class Members

All Classes Namespaces Files Functions Variables Typedefs Enumerations Enumerator Friends Macros Pages

- stan
- gm
- singleton\_argument

Public Member Functions |
Protected Member Functions |
Protected Attributes |
List of all members

stan::gm::singleton\_argument< T > Class Template Reference

`#include <singleton_argument.hpp>`

Inheritance diagram for stan::gm::singleton\_argument< T >:

|  |  |
| --- | --- |
| Public Member Functions | |
|  | singleton\_argument () |
|  | |
|  | singleton\_argument (const std::string name) |
|  | |
| bool | parse\_args (std::vector< std::string > &args, std::ostream \*out, std::ostream \*err, bool &help\_flag) |
|  | |
| virtual void | probe\_args (argument \*base\_arg, std::stringstream &s) |
|  | |
| void | find\_arg (std::string name, std::string prefix, std::vector< std::string > &valid\_paths) |
|  | |
| T | value () |
|  | |
| bool | set\_value (const T &value) |
|  | |
| std::string | print\_value () |
|  | |
| std::string | print\_valid () |
|  | |
| bool | is\_default () |
|  | |
| Public Member Functions inherited from stan::gm::valued\_argument | |
| virtual void | print (std::ostream \*s, const int depth, const std::string prefix) |
|  | |
| virtual void | print\_help (std::ostream \*s, const int depth, const bool recurse=false) |
|  | |
| Public Member Functions inherited from stan::gm::argument | |
|  | argument () |
|  | |
|  | argument (const std::string name) |
|  | |
| virtual | ~argument () |
|  | |
| std::string | name () const |
|  | |
| std::string | description () const |
|  | |
| virtual argument \* | arg (const std::string name) |
|  | |
| int | compute\_indent (const int depth) |
|  | |

|  |  |
| --- | --- |
| Protected Member Functions | |
| virtual bool | is\_valid (T value) |
|  | |

|  |  |
| --- | --- |
| Protected Attributes | |
| std::string | \_validity |
|  | |
| T | \_value |
|  | |
| T | \_default\_value |
|  | |
| bool | \_constrained |
|  | |
| T | \_good\_value |
|  | |
| T | \_bad\_value |
|  | |
| Protected Attributes inherited from stan::gm::valued\_argument | |
| std::string | \_default |
|  | |
| std::string | \_value\_type |
|  | |
| Protected Attributes inherited from stan::gm::argument | |
| std::string | \_name |
|  | |
| std::string | \_description |
|  | |
| int | indent\_width |
|  | |
| int | help\_width |
|  | |

|  |  |
| --- | --- |
| Additional Inherited Members | |
| Static Public Member Functions inherited from stan::gm::argument | |
| static void | split\_arg (const std::string &arg, std::string &name, std::string &value) |
|  | |

## Detailed Description

### template<typename T> class stan::gm::singleton\_argument< T >

Definition at line 44 of file singleton\_argument.hpp.

## Constructor & Destructor Documentation

template<typename T >

|  |  |  |  |  |
| --- | --- | --- | --- | --- |
| stan::gm::singleton\_argument< T >::singleton\_argument | ( |  | ) |  |

inline

Definition at line 48 of file singleton\_argument.hpp.

template<typename T >

|  |  |  |  |  |  |
| --- | --- | --- | --- | --- | --- |
| stan::gm::singleton\_argument< T >::singleton\_argument | ( | const std::string | *name* | ) |  |

inline

Definition at line 54 of file singleton\_argument.hpp.

## Member Function Documentation

template<typename T >

|  |  |  |  |
| --- | --- | --- | --- |
| void stan::gm::singleton\_argument< T >::find\_arg | ( | std::string | *name*, |
|  |  | std::string | *prefix*, |
|  |  | std::vector< std::string > & | *valid\_paths* |
|  | ) |  |  |

inlinevirtual

Reimplemented from stan::gm::argument.

Definition at line 116 of file singleton\_argument.hpp.

template<typename T >

|  |  |  |  |  |
| --- | --- | --- | --- | --- |
| bool stan::gm::singleton\_argument< T >::is\_default | ( |  | ) |  |

inlinevirtual

Implements stan::gm::valued\_argument.

Definition at line 144 of file singleton\_argument.hpp.

template<typename T >

|  |  |  |  |  |  |
| --- | --- | --- | --- | --- | --- |
| virtual bool stan::gm::singleton\_argument< T >::is\_valid | ( | T | *value* | ) |  |

inlineprotectedvirtual

Definition at line 152 of file singleton\_argument.hpp.

template<typename T >

|  |  |  |  |
| --- | --- | --- | --- |
| bool stan::gm::singleton\_argument< T >::parse\_args | ( | std::vector< std::string > & | *args*, |
|  |  | std::ostream \* | *out*, |
|  |  | std::ostream \* | *err*, |
|  |  | bool & | *help\_flag* |
|  | ) |  |  |

inlinevirtual

Reimplemented from stan::gm::argument.

Definition at line 59 of file singleton\_argument.hpp.

template<typename T >

|  |  |  |  |  |
| --- | --- | --- | --- | --- |
| std::string stan::gm::singleton\_argument< T >::print\_valid | ( |  | ) |  |

inlinevirtual

Implements stan::gm::valued\_argument.

Definition at line 140 of file singleton\_argument.hpp.

template<typename T >

|  |  |  |  |  |
| --- | --- | --- | --- | --- |
| std::string stan::gm::singleton\_argument< T >::print\_value | ( |  | ) |  |

inlinevirtual

Implements stan::gm::valued\_argument.

Definition at line 136 of file singleton\_argument.hpp.

template<typename T >

|  |  |  |  |
| --- | --- | --- | --- |
| virtual void stan::gm::singleton\_argument< T >::probe\_args | ( | argument \* | *base\_arg*, |
|  |  | std::stringstream & | *s* |
|  | ) |  |  |

inlinevirtual

Reimplemented from stan::gm::argument.

Definition at line 98 of file singleton\_argument.hpp.

template<typename T >

|  |  |  |  |  |  |
| --- | --- | --- | --- | --- | --- |
| bool stan::gm::singleton\_argument< T >::set\_value | ( | const T & | *value* | ) |  |

inline

Definition at line 126 of file singleton\_argument.hpp.

template<typename T >

|  |  |  |  |  |
| --- | --- | --- | --- | --- |
| T stan::gm::singleton\_argument< T >::value | ( |  | ) |  |

inline

Definition at line 124 of file singleton\_argument.hpp.

## Member Data Documentation

template<typename T >

T stan::gm::singleton\_argument< T >::\_bad\_value

protected

Definition at line 160 of file singleton\_argument.hpp.

template<typename T >

bool stan::gm::singleton\_argument< T >::\_constrained

protected

Definition at line 157 of file singleton\_argument.hpp.

template<typename T >

T stan::gm::singleton\_argument< T >::\_default\_value

protected

Definition at line 155 of file singleton\_argument.hpp.

template<typename T >

T stan::gm::singleton\_argument< T >::\_good\_value

protected

Definition at line 159 of file singleton\_argument.hpp.

template<typename T >

std::string stan::gm::singleton\_argument< T >::\_validity

protected

Definition at line 151 of file singleton\_argument.hpp.

template<typename T >

T stan::gm::singleton\_argument< T >::\_value

protected

Definition at line 154 of file singleton\_argument.hpp.

---

The documentation for this class was generated from the following file:

- src/stan/gm/arguments/singleton\_argument.hpp


---

[ Stan Home Page ]

*© 2011–2014,
Stan Development Team.*
